# Work-Home Interference, Perceived Total Workload, and the Risk of Future Sickness Absence Due to Stress-Related Mental Diagnoses Among Women and Men: a Prospective Twin Study

**DOI:** 10.1007/s12529-017-9669-9

**Published:** 2017-06-21

**Authors:** Pia Svedberg, Lisa Mather, Gunnar Bergström, Petra Lindfors, Victoria Blom

**Affiliations:** 1 Division of Insurance Medicine, Department of Clinical Neuroscience, Karolinska Institutet, Berzeliusv. 3, 171 77 Stockholm, Sweden; 2 Division of Intervention and Implementation Research, The Institute of Environmental Medicine, Karolinska Institutet, Stockholm, Sweden; 3 Department of Psychology, Stockholm University, Stockholm, Sweden; 4 The Swedish School of Sport and Health Sciences, Stockholm, Sweden

**Keywords:** Sick leave, Work-home interference, Work disability, Twins, Work load, Gender

## Abstract

**Purpose:**

Work-home interference has been proposed as an important explanation for sickness absence (SA). Previous studies show mixed results, have not accounted for familial factors (genetics and shared everyday environment), or investigated diagnosis specific SA. The aim was to study whether work-home interference and perceived total workload predict SA due to stress-related mental diagnoses, or SA due to other mental diagnoses, among women and men, when adjusting for various confounders and familial factors.

**Methods:**

This study included 11,916 twins, 19–47 years (49% women). Data on work-to-home and home-to-work conflicts, perceived total workload, and relevant confounders were derived from a 2005 survey, and national register data on SA spells until 2013 were obtained. Odds ratios (ORs) with 95% confidence intervals (CIs) were calculated. Discordant twin pair design was applied to adjust for familial factors.

**Results:**

Each one unit increase in work-to-home and home-to-work conflicts, and perceived total workload was associated with higher odds for SA due to stress-related mental diagnoses and to SA due to other mental diagnoses among women, when adjusting for sociodemographic factors (ORs 1.15–1.31). Including health or familial factors, no associations remained. For men, each one unit increase in work-to-home conflicts was associated with higher odds for SA due to stress-related diagnoses (ORs 1.23–1.35), independently of confounders.

**Conclusion:**

Work-to-home conflict was independently associated with future SA due to stress-related diagnoses among men only. Health- and work-related factors seem to be important confounders when researching work-home interference, perceived total workload, and SA. Not including such confounders involves risking drawing incorrect conclusions. Further studies are needed to confirm sex differences and whether genetic factors are important for the associations studied.

## Background

Changes in work and home domains involve more people struggling to combine work and family life. Unequal distribution of home duties along with a high total workload has been suggested to explain why women tend to report negative work-home balance to a higher degree than men [1, 2]. However, an imbalance between work and home responsibilities has been associated with sub-optimal health in both women and men [2–5]. Considering this, the interference between work and family life has been suggested as an important explanation for sickness absence (SA) [2, 6–10], alongside factors relating to individuals’ health, work environment, sociodemographic, and lifestyle factors [11].

Despite an increased focus on SA, knowledge of specific risk factors, including work-home interference, remains inconsistent. Most research focuses on overall SA, while fewer studies include SA due to specific diagnoses. Currently, mental disorders that include the ICD-10 diagnosis F43, i.e., reaction to severe stress and adjustment disorders (from now referred to as stress-related mental disorders), are the most common reasons for SA, especially among women and younger individuals in Sweden and in other European countries [12, 13]. However, many studies of SA risk factors are hampered by cross-sectional designs or selection biases relating to health factors or family background. Various health conditions including mental disorders are influenced by genetic factors, which in turn may influence the risk of experiencing stress. Further, previous studies have shown that SA is moderately heritable. But, a population-based twin-setting including twins sharing their genes and having grown up in the same family allows controlling for genetic and shared environmental (familial) confounders. No previous study has used this design to investigate work-home interference as a risk factor for SA.

Negative health outcomes of work-home interference may result of negative spillover effects due to situations including an inter-role conflict, i.e., being involved at work may put strain on the family role, or vice versa [14, 15]. Consequently, two types of work-home interference may follow: work-to-home conflict referring to work-role demands having an unfavorable impact on the home and family roles and home-to-work conflict which refers to demands at home having an unfavorable impact on individuals’ work roles [16, 17]. Many studies report spillover effects but also find that dispositional factors and work characteristics are important for work-home interference [18–20].

Regarding SA, studies are few and findings mixed. In one study, work-to-home conflict was associated with almost threefold higher odds of SA among men in higher socioeconomic strata, while no such association emerged for women [8]. Another study found that women reporting high work-to-home conflict were at higher risk for SA [6]. Also, recent research found that gender, age, and family situation, including having children, play a role for the associations [6, 21]. Others have found that home-to-work conflict is associated with long SA duration (>10 SA days) and high SA frequency in both women and men, also when adjusting for sociodemographic factors, health indicators, and psychosocial factors [7]. Jansen and colleagues [9] found a clear association between home-to-work conflicts but not for work-to-home conflict and SA.

With previous studies showing that work-home interference is associated with sub-optimal health, mental disorders, and burnout [4, 5, 22, 23], it seems reasonable to assume that work-home interference is a risk factor for SA due to stress-related mental diagnoses or other mental diagnoses. However, with previous research on work-home interference having focused on the association to SA in general, using cross-sectional or prospective designs with shorter follow-ups, it is unclear whether effects differ between SA diagnoses when using a follow-up time of several years.

So far, there are no twin studies of work-home interference or the perception of total workload and the risk of future SA. But, a recent twin study showed that both work-to-home and home-to-work conflicts were associated with burnout and that genetic factors seemed to confound the association between home-to-work conflict and burnout [4]. Another study identified high job demands and job strain as risk factors for SA due to mental disorders, with familial factors seeming important for the association between job support and incident SA [24]. Moreover, recent research indicates that childhood experiences of the family influence how adults perceive work strain and demands [25]. Furthermore, twin studies have shown the importance of genetics for SA [26, 27] but also for other individual factors. This includes associations with abilities relevant for handling work-home balance and experiences of a high workload, such as coping behaviors [28] and cognitive resources [29, 30]. Additionally, a longitudinal study found that work-home interference is fairly stable throughout life and not only limited to the early working career [31]. This suggests that some dispositional factors, such as personality, which is highly influenced by genetics, could be involved. Moreover, it is well-known that genetics play a role in mental disorders [32] underlying work disability even though one twin study has suggested that the association between internalizing mental disorders and SA is influenced by unique environmental factors rather than by genetics [33]. But, taking together previous findings makes it reasonable to assume that the associations between work-home interference and SA are influenced by familial factors in addition to psychosocial, health, or work-related factors.

The aim of the present prospective study was to investigate whether work-to-home and home-to-work conflicts and perceived total workload are risk factors for future SA due to stress-related mental disorders (ICD-10, diagnosis F43), and SA due to other mental disorders, among women and men, also when adjusting for confounders including familial (genetics and shared environment) factors.

## Methods

### Study Population

**Fig. 1:**
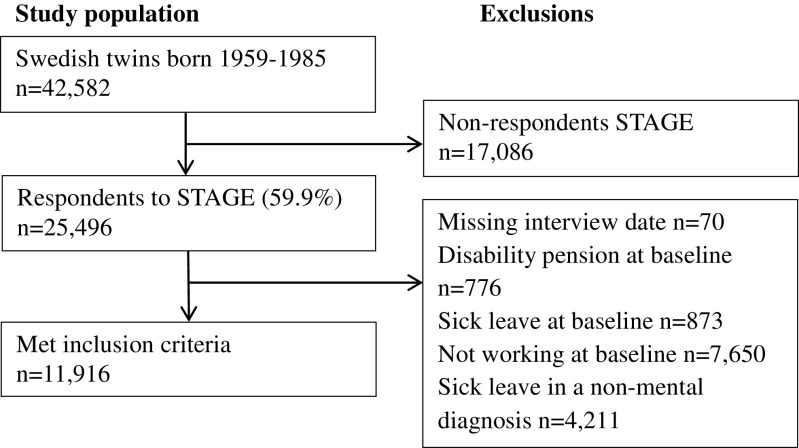
Flowchart of the study sample: inclusion and exclusion criteria

The source population consisted of 25,496 twins born between 1959 and 1985 of the Swedish Twin project of Disability pension and Sickness absence (STODS) who participated in the Study of Twin Adults: Genes and Environment (STAGE) web-based survey conducted by the Swedish Twin Registry in 2005 [34]. The present study investigated work-home interference and perceived total workload in association to SA. So, only data from working individuals were included. Individuals being disability pensioned at the time of interview or only having SA spells due to non-mental diagnoses at follow-up were excluded. The final study sample included 11,916 twins (49% women), aged 19–47 (mean = 35.4, *SD* 6.8) (see Fig. 1 for inclusion criteria). Of these, 2385 were complete pairs, 942 monozygotic (MZ) pairs, 723 same-sex dizygotic (DZ) pairs, and 720 opposite-sex pairs. Also 7146 single twins were included, i.e., the twin sibling did not respond to STAGE, or belonged to pairs of unknown zygosity, or were excluded based on the above criteria. For details on zygosity determination in the Swedish Twin Registry, see Lichtenstein et al. [34].

### Outcomes and Follow-Up Time

SA data were obtained from the National Social Insurance Agency MicroData for Analyses of Social insurance database (MiDAS) and linked to each individual using the Swedish ten-digit personal identification number. All individuals in Sweden above the age of 16, with an income from work or unemployment benefits, can receive sickness benefits paid by the Social Insurance Agency when disease or injury has caused reduced work capacity. Employees receive sick pay from their employers during the first 14 days after a qualifying day (usually more qualifying days for self-employed) without benefits. Diagnosis-specific SA during follow-up was defined based on ICD-10 codes [35]. SA was operationalized as having at least one incident spell lasting longer than 14 days during follow-up i.e., between the date of STAGE survey response (varying between 11/01/2004 to 04/21/2006) and 12/31/2013. Two outcome variables were created; *SA due to stress-related diagnoses* (ICD-10 F43) and *SA due to other mental diagnoses* (other diagnoses in the F-chapter, except for F43 episodes during follow-up). Hierarchy was applied and priority was given to spells in stress-related mental diagnoses, followed by other mental diagnoses. No SA spell during follow-up was used as reference.

### Exposures

Work-home interference was measured with the following two questions: “Do the demands in your work affect your home and family life in a negative way” (*work-to-home conflict)* and “Do the demands in your home/family affect your work in a negative way” (*home-to-work conflict*). These items were originally developed for the General Nordic Questionnaire for psychological and social factors at work (QPSNordic) [36].

*Perceived total workload* was assessed using the question “Do you have difficulties getting sufficient time for both work and personal life?” It measures an individual’s perception of the total workload, which is distinct from the actual amount of work.

For all three variables, the STAGE used a four-point response format: 1 = always, 2 = sometimes, 3 = almost never, and 4 = never. Responses were reversed with high scores indicating more conflict or a higher perceived workload.

### Confounders

This study includes factors previously associated with work-home interference and SA.

#### Sociodemographic Factors

*Age* was included as a continuous variable derived by subtracting the date of response to STAGE from the birthdate. *Sex* was dichotomous (women and men). The highest level of *education* was categorized into three groups (1) elementary school, (2) vocational school, and (3) university degree (military school and vocational university were included in category 3 and residential college for adult education in category 2). *Marital status* was grouped into married/cohabiting or not. *Living with children* was measured as a dichotomous variable, stating whether an individual had children living at home or not.

#### Work- and Health-Related Factors

*Work status* was measured as a dichotomous variable with an individual reporting working full-time, being full-time employed, or full-time self-employed, or a combination of part-time employment and part-time self-employment (working full-time), and others i.e., not working full-time. The Swedish translation [37] of the Karasek and Theorell [38] questionnaire was used to assess *job demands, control, and support*. Responses were given on a four-point Likert scale, from 1 = do not agree to 4 = agree entirely. Mean scores were calculated of job demands, control, and support and used as continuous variables. *Self-rated health (SRH)* was asked for in STAGE with the question “How would you rate your general health status?” with response alternatives excellent, good, moderate, fairly poor, and poor. With few responses in the lowest categories, ‘fairly poor’ and ‘poor’ were collapsed into one category. *Previous sick leave* was based on MiDAS data (episodes of SA > 14 days in a row) between 2003 and STAGE response (approximately a 2-year period) (yes/no).

### Statistical Analyses

Logistic regression analyses were used to assess odds ratios (ORs) with 95% confidence intervals (CIs), stratified by sex to assess the associations between work-to-home conflict, home-to-work conflict, perceived total workload, and SA. The responses “do not know/do not want to answer” were treated as missing values. Analyses were adjusted for the study sample including twin pairs rather than independent individuals by using the clustered robust standard error. In the analysis of the whole sample, covariates were entered in three blocks: first sociodemographic factors (age, education, and marital status) were entered (model 1), then living with children, work status, job demands, control, and support were entered (model 2), and finally previous history of SA and SRH were added (model 3). An additional analysis combining women and men, adjusting for age and sex, was also conducted, and we tested the interaction effects with sex. Co-twin control (conditional logistic regression) analyses based on same-sex discordant MZ and DZ twin pairs were conducted to adjust for familial (genetics and shared family environment) confounding [39, 40]. A twin pair was treated as discordant if only one twin of a pair had incident SA during follow-up. In co-twin control analyses, twins in a pair are optimally matched on genetics (MZ 100% and DZ on average 50%) and shared environmental factors (100%) when reared together, and for age and sex. An influence of familial factors is indicated if an association found in the whole sample disappears or changes considerably in the analyses of discordant twin pairs. If the association is stronger in DZ than MZ pairs, genetics rather than shared environmental factors are of importance, while familial factors will be assumed to play a minor role if the association is found in the analyses of both the whole sample and of discordant twin pairs. Co-twin analyses were conducted both stratified by sex (MZ and DZ pairs combined) and stratified by zygosity. In addition, an unconditional logistic regression analysis of all the 418 individuals belonging to SA discordant twin pairs was conducted. All analyses were conducted using STATA IC 12.1.

## Results

Table 1 presents descriptive statistics for the whole sample (*n* = 11,916), by sex and SA status during follow-up. More women than men had SA spells during follow-up. Among women, SA due to stress-related mental diagnoses was more common than SA due to other mental diagnoses. Among men, SA due to stress-related and SA due to other mental diagnoses were equally common. Table 2 shows results of the logistic regression analyses stratified by sex, which revealed that for women, each one unit increase in work-to-home and home-to-work conflicts and perceived total workload were associated with higher odds for future SA due to stress-related mental diagnoses and to SA due to other mental diagnoses, also when adjusting for sociodemographic factors (ORs 1.15–1.31). The associations between work-to-home conflict, perceived total workload, and SA due to stress-related mental diagnoses were non-significant after adjusting for work-related factors and living with children. Similar results emerged for the associations between home-to-work conflict, perceived total workload, and SA due to other mental diagnoses. When adjusting for previous SA history and SRH, no associations remained.

**Table 1:** Frequencies of exposures and covariates for 11,916 Swedish twin individuals stratified by sickness absence (SA) status during follow-up and sex

	No SA		SA due to stress-related mental diagnoses		SA due to other mental diagnoses	
	Women (*n* = 4707)	Men (*n* = 5697)	Women (*n* = 656)	Men (*n* = 197)	Women (*n* = 459)	Men (*n* = 200)
Exposures	*n* (%)/mean (SD)	*n* (%)/mean (SD)	*n* (%)/mean (SD)	*n* (%)/mean (SD)	*n* (%)/mean (SD)	*n* (%)/mean (SD)
Work-to-home conflict (WHC)						
1. never/almost never	1594 (33.9)	2060 (36.2)	164 (25.0)	53 (26.9)	132 (28.8)	68 (34.0)
2. Seldom	985 (20.9)	1294 (22.7)	132 (20.1)	36 (18.3)	86 (18.7)	43 (21.5)
3. Sometimes	1462 (31.0)	1701 (29.8)	249 (38.0)	78 (39.6)	155 (33.8)	59 (29.5)
4. Often	243 (5.2)	336 (5.9)	48 (7.3)	18 (9.1)	55 (12.0)	17 (8.5)
Missing	423 (9.0)	306 (5.4)	63 (9.6)	12 (6.1)	31 (6.7)	13 (6.5)
WHC (mean, 1–4)	2.1 (1.0)	2.1 (1.0)	2.3 (1.0)	2.3 (1.0)	2.3 (1.0)	2.1 (1.0)
Home-to-work conflict (HWC)						
1. Never/almost never	2476 (52.6)	3041 (53.4)	292 (44.5)	91 (46.2)	235 (51.2)	109 (54.5)
2. Seldom	1119 (23.8)	1411 (24.8)	164 (25.0)	52 (26.4)	97 (21.1)	40 (20.0)
3. Sometimes	634 (13.5)	865 (15.2)	121 (18.5)	40 (20.3)	82 (17.9)	35 (17.5)
4. Often	53 (1.1)	71 (1.2)	16 (2.4)	3 (1.5)	14 (3.0)	4 (2.0)
Missing	425 (9.0)	309 (5.4)	63 (9.6)	11 (5.6)	31 (6.8)	12 (6.0)
HWC (mean, 1–4)	1.6 (0.8)	1.6 (0.8)	1.8 (0.9)	1.8 (0.8)	1.7 (0.9)	1.7 (0.9)
Perceived total workload (PTW)						
1. Never/almost never	917 (19.5)	1236 (21.7)	102 (15.5)	39 (19.8)	87 (19.0)	43 (21.5)
2. Seldom	697 (14.8)	966 (17.0)	78 (11.9)	28 (14.2)	47 (10.2)	31 (15.5)
3. Sometimes	1758 (37.4)	2014 (35.3)	241 (36.7)	60 (30.5)	169 (36.8)	66 (33.0)
4. Often	919 (19.5)	1181 (20.7)	171 (26.1)	59 (29.9)	126 (27.5)	47 (23.5)
Missing	416 (8.8)	300 (5.3)	64 (9.8)	11 (5.6)	30 (6.5)	13 (6.5)
PTW (mean, 1–4)	2.6 (1.0)	2.6 (1.1)	2.8 (1)	2.8 (1.1)	2.8 (1.1)	2.6 (1.1)
Covariates	*n* (%)/mean (SD)	*n* (%)/mean (SD)	*n* (%)/mean (SD)	*n* (%)/mean (SD)	*n* (%)/mean (SD)	*n* (%)/mean (SD)
Age (range 19–47)	35.6 (6.9)	35.1 (6.8)	35.9 (6.4)	36.1 (6.7)	34.9 (7.1)	36.1 (6.9)
Education						
Elementary/vocational	1955 (41.5)	2814 (49.4)	277 (42.2)	97 (49.2)	228 (49.7)	111 (55.5)
University	2475 (52.6)	2629 (46.1)	325 (49.6)	87 (44.2)	187 (40.7)	78 (39.0)
Missing	277 (5.9)	254 (4.5)	54 (8.2)	13 (6.6)	44 (9.6)	11 (5.5)
Marital status						
Married/cohabiting	3426 (72.8)	3966 (69.6)	491 (74.8)	135 (68.5)	319 (69.5)	124 (62.0)
Other	1273 (27.0)	1726 (30.3)	165 (25.2)	61 (31.0)	140 (30.5)	76 (38.0)
Missing	8 (0.2)	5 (0.1)	0	1 (0.5)	0	0
Living with children						
Yes	2811 (59.7)	2806 (49.3)	427 (65.1)	95 (48.2)	274 (59.7)	100 (50.0)
No	1896 (40.3)	2891 (50.7)	229 (34.9)	102 (51.8)	185 (40.3)	100 (50.0)
Work full time						
Yes	3393 (72.1)	5378 (94.4)	488 (74.4)	189 (95.9)	322 (70.2)	184 (92.0)
No	1314 (27.9)	319 (5.6)	168 (25.6)	8 (4.1)	137 (29.8)	16 (8.0)
Job demands (mean, 1–4)	2.5 (0.6)	2.5 (0.6)	2.7 (0.6)	2.6 (0.6)	2.6 (0.6)	2.6 (0.6)
Control (mean, 1–4)	3 (0.6)	3.1 (0.5)	3 (0.6)	3.1 (0.6)	2.9 (0.6)	3.1 (0.6)
Support (mean, 1–4)	3.4 (0.5)	3.4 (0.5)	3.3 (0.5)	3.3 (0.5)	3.3 (0.5)	3.3 (0.5)
Previous sick leave						
Yes	669 (14.2)	409 (7.2)	229 (34.9)	37 (18.8)	183 (39.9)	51 (25.5)
No	4038 (85.8)	5288 (92.8)	427 (65.1)	160 (81.2)	276 (60.1)	149 (74.5)
Self-rated health						
Excellent	1639 (34.8)	2038 (35.8)	158 (24.1)	52 (26.4)	83 (18.1)	48 (24.0)
Good	2342 (49.8)	2762 (48.5)	333 (50.7)	106 (53.8)	217 (47.3)	84 (42.0)
Moderate	567 (12.0)	672 (11.8)	137 (20.9)	31 (15.7)	112 (24.4)	43 (21.5)
Fairly poor/poor	78 (1.7)	55 (0.9)	15 (2.3)	5 (2.6)	32 (7.0)	16 (8.0)
Missing	81 (1.7)	170 (3.0)	13 (2.0)	3 (1.5)	15 (3.2)	9 (4.5)

**Table 2:** Odds ratios (OR) with 95% confidence intervals (CI) for work-home interference and perceived total workload as predictors for sickness absence (SA) due to stress-related diagnoses and SA due to other mental diagnoses among 11,916 Swedish twins and discordant twin pair analysis (co-twin) among same-sex monozygotic (MZ) and dizygotic (DZ) twin pairs (116 discordant for SA due to stress-related and 93 discordant for SA due to other mental diagnoses). Analyses stratified by sex

	SA due to stress-related diagnoses		SA due to other mental disorders	
	OR	95% CI	OR	95% CI
Women				
Work-to-home conflict				
Crude	*1.27*	1.16–1.38	*1.27*	1.14–1.41
Model 1	*1.26*	1.14–1.38	*1.31*	1.17–1.47
Model 2	1.12	1.00–1.24	*1.28*	1.12–1.46
Model 3	1.07	0.95–1.19	1.15	1.00–1.32
Co-twin model (MZ + DZ)	1.17	0.77–1.78	1.03	0.66–1.59
Home-to-work conflict				
Crude	*1.29*	1.16–1.43	*1.19*	1.05–1.35
Model 1	*1.28*	1.14–1.42	*1.20*	1.05–1.38
Model 2	*1.18*	1.04–1.32	1.15	0.99–1.33
Model 3	1.12	0.99–1.27	1.05	0.90–1.22
Co-twin model (MZ + DZ)	0.92	0.65–1.31	1.05	0.67–1.64
Perceived total workload				
Crude	*1.19*	1.09–1.30	*1.15*	1.04–1.28
Model 1	*1.20*	1.09–1.31	*1.17*	1.06–1.30
Model 2	1.07	0.97–1.19	1.12	0.99–1.26
Model 3	1.03	0.93–1.15	1.03	0.91–1.16
Co-twin model (MZ + DZ)	1.20	0.86–1.67	0.84	0.55–1.27
Men				
Work-to-home conflict				
Crude	*1.32*	1.14–1.54	1.08	0.93–1.26
Model 1	*1.35*	1.15–1.57	1.09	0.92–1.28
Model 2	*1.25*	1.05–1.49	1.05	0.87–1.26
Model 3	*1.23*	1.03–1.47	0.93	0.77–1.12
Co-twin model (MZ + DZ)	1.54	0.91–2.61	0.78	0.39–1.55
Home-to-work conflict				
Crude	*1.22*	1.03–1.45	1.04	0.86–1.26
Model 1	1.20	1.00–1.45	1.04	0.85–1.28
Model 2	1.13	0.92–1.39	1.02	0.82–1.27
Model 3	1.08	0.88–1.33	0.91	0.72–1.15
Co-twin model (MZ + DZ)	1.18	0.65–2.17	0.85	0.48–1.49
Perceived total workload				
Crude	1.16	1.00–1.35	1.04	0.90–1.20
Model 1	1.16	0.99–1.36	1.04	0.90–1.21
Model 2	1.08	0.92–1.28	1.01	0.85–1.20
Model 3	1.07	0.91–1.27	0.98	0.83–1.17
Co-twin model (MZ + DZ)	1.03	0.65–1.61	1.05	0.68–1.62

Statistically significant ORs in italics. Model 1: adjusted for age, education, and marital status; model 2: adjusted for age, education, marital status, living with children, work full time, job demands, control, and support; model 3: adjusted for age, education, marital status, living with children, work full time, job demands, control, support, previous sick leave, and self-rated health.

*MZ* monozygotic, *DZ* dizygotic

For men, each one-unit increase in work-to-home conflicts was associated with higher odds for SA due to stress-related diagnoses, also after adjusting for all covariates (ORs in models 1–3; 1.23–1.35). Only the crude model showed an association between home-to-work conflicts and SA due to stress-related diagnoses for men. No associations emerged between the exposures and SA due to other mental disorders.

Analyzing women and men together, adjusting for age and sex, each one unit increase in all three exposures were significantly associated with higher odds (ORs 1.12–1.28) for future SA due to stress-related or other mental diagnoses (see Table 3). We found no statistically significant interaction effects with sex.

**Table 3:** Odds ratios (OR) with 95% confidence intervals (CI) for work-home interference and perceived total workload as predictors for sickness absence (SA) due to stress-related or other mental diagnoses; for the whole sample (11,916 twins) and of the discordant (co-twin) same-sex twin pairs (116 discordant for SA due to stress-related and 93 discordant for SA due to other mental diagnoses) by zygosity group

	Whole sample^a^	Co-twin analysis		
	OR (95% CI)	Discordant twin pairs^b^ OR (95% CI)	DZ OR (95% CI)	MZ OR (95% CI)
SA due to stress-related mental diagnoses				
Work-to-home conflict	*1.28 (1.18–1.38)*	1.22 (0.96–1.56)	1.47 (0.95–2.28)	1.13 (0.69–1.84)
Home-to-work conflict	*1.26 (1.15–1.38)*	0.99 (0.76–1.29)	1.31 (0.83–2.06)	0.72 (0.47–1.11)
Perceived total workload	*1.18 (1.09–1.27)*	1.10 (0.88–1.37)	1.29 (0.88–1.89)	1.00 (0.69–1.46)
SA due to other mental diagnoses				
Work-to-home conflict	*1.21 (1.11–1.33)*	0.97 (0.76–1.22)	1.20 (0.73–1.96)	0.68 (0.38–1.21)
Home-to-work conflict	*1.15 (1.03–1.28)*	1.00 (0.70–1.42)	1.22 (0.73–2.04)	0.79 (0.49–1.26)
Perceived total workload	*1.12 (1.03–1.22)*	0.95 (0.74–1.21)	1.05 (0.73–1.52)	0.73 (0.44–1.21)

Statistically significant ORs in italics

*MZ* monozygotic, *DZ* dizygotic

^a^Women and men combined, adjusted for sex and age

^b^Unconditional logistic regression analysis of all the 232 and 186 individuals, respectively, belonging to SA discordant twin pairs

Discordant twin pair analyses (see Table 3) showed no statistically significant associations between work-to-home or home-to-work conflicts or perceived total workload and SA due to stress-related or other mental diagnoses for MZ or DZ twin pairs. However, for discordant DZ twin pairs estimates followed those of the whole sample but with less precision. This suggests that genetic factors may be of importance for the studied associations. Results of the unconditional analysis of the 418 individuals belonging to SA discordant pairs showed non-significant estimates for the associations between work-to-home and home-to-work conflicts, perceived total workload, and SA due to stress-related or other mental diagnoses. Estimates were as expected in between the estimates of DZ and MZ pairs (conditional models) and with less precision than estimates of the whole cohort (Table 3). For comparative purposes, Table 2 presents ORs for MZ and DZ discordant twin pairs combined but stratified by sex; none of the estimates reached statistical significance.

## Discussion

This prospective twin cohort study of 11,916 working-age women and men provided a unique opportunity to investigate work-home interference and perceived total workload as risk factors for SA due to stress-related or due to other mental diagnoses. We took advantage of a discordant twin pair design to account for familial (genetics and shared environment) confounding. The associations were also studied while adjusting for several relevant confounders. Specifically, we found no significant associations between work-home interference, perceived total workload, and SA due to stress-related or other mental diagnoses for women when adjusting for various confounders. However, among men, work-to-home conflict was associated with SA due to stress-related mental diagnoses, independently of the confounding factors. In line with expectations, various confounders including work or health aspects and perhaps genetics seemed to explain most of the associations.

Even though differences in employment rates between women and men have decreased in many countries, traditional gender patterns regarding, for example, responsibility for the household and children remain. Moreover, the labor market is gendered, as is work disability. This means that the potential sex differences found in the present study may reflect an unequal division of home responsibilities and/or unequal opportunities in working life. However, these results need to be replicated in studies including larger samples before drawing any firm conclusions regarding possible sex differences. Previous research suggests that the unequal sharing of household responsibilities is associated with negative health outcomes, especially among women [2]. Our results suggest that, for women, the associations between work-to-home and home-to-work conflicts, perceived total workload, and SA due to stress-related or due to other mental diagnoses were similar. Also, these associations were explained either by work- or health-related factors, and potentially genetics with these results being less clear. For men, only work-to-home conflict was associated to SA due to stress-related mental diagnoses and independently of the confounders. This follows previous research showing sex differences in the association between work-to-home conflict and SA in general [8]. Thus, the results indicate that the gendered work and family life may manifest differently for women and men in relation to SA. For men, this involves excessive demands at work, and for women, it is most likely the balancing of excessive demands both at home and at work. But for women, the importance of ill-health and/or genetic vulnerability for diseases or factors such as e.g. neuroticism and conscientiousness [41] is pronounced. Many studies show that genetic factors are important for mental disorders [32]. Women are more often diagnosed with mental disorders and consequently also on sick leave due to such disorders to a higher degree then men [12]. Most likely, the genetic influences in the present study reflect health factors, since the previous history of SA and SRH confounds the results of associations in the whole cohort. More research, with larger samples, is needed to identify explanatory factors for the association between work-to-home conflict and SA due to stress-related mental diagnoses among men.

### Strengths and Limitations

This study has several strengths including the large and genetically informed population-based sample, objective SA data of high quality with complete coverage from a national register, and a prospective cohort design. Also, extensive survey data including validated measures of work-home interference and relevant confounders were available. Using single-item exposures may introduce measurement error. However, the items included in the present analyses were originally developed for the General Nordic Questionnaire for Psychological and Social factors at work (QPSNordic) and the psychometric testing of this questionnaire suggests its good qualities for assessing health-related factors at work [36], and the items have been widely used in studies of work-home interference and various outcomes (e.g., [8]) and are in accordance with those of Frone and colleagues [17]. A unique strength includes the possibility to control for familial confounding using the discordant twin pairs i.e., to determine whether an association is likely to reflect a causal relationship [39]. Here, we found some support for a direct effect of work-to-home conflict on future SA due to stress-related mental diagnoses but only among men. However, any interpretation of the results needs to acknowledge study limitations. First, questionnaire data always include some missing data. Yet, in the final sample, the amount of missing data based on self-report measures and included confounders were low. Second, without survey follow-ups, exposures were only assessed at a single time-point. Consequently, it is unclear whether reports of work-home interference or confounding factors change, and if such changes influence the risk of SA. Further, only twins, aged 19–47, born in Sweden were included which reduces generalizability to other groups such as immigrants, other countries, and older adults. Also, the issue of whether physicians are able to reliably distinguish between F43 and other ICD diagnoses of mental disorders should be considered. For this study, only one main SA diagnosis was available meaning that misclassification or comorbidity may be present. Recent findings also show changes in the primary diagnosis to a diagnosis from another diagnostic chapter during the same episode; this happened to 7.1% of women cases and 6.6% of men cases. However, such a change of the primary diagnosis was least common among those initially sick-listed for mental and musculoskeletal disorders [42]. The patterns of diagnostic changes were similar for women and men. Yet, changes within a diagnosis chapter remain to be investigated. Finally, we cannot rule out the influence of familial factors on the associations studied; estimates of the discordant twin pair analyses had lower precision and need to be interpreted with caution. Additional studies with larger samples are needed to confirm or reject the influence of genetic factors on the association between work-home interference and SA.

### Conclusions

This study suggests potential sex differences in the associations between work-home interference, perceived total workload, and SA due to stress-related and other mental diagnoses, also with respect to influential factors. For men, an independent association emerged between work-to-home conflict and SA due to stress-related mental diagnoses. Work, health, and also potentially genetic factors seem to be important confounders, particularly among women. Importantly, disregarding health status and work factors may result in erroneous conclusions regarding the true effect of work-home interference on future SA.
